# Preference and Prey Switching in a Generalist Predator Attacking Local and Invasive Alien Pests

**DOI:** 10.1371/journal.pone.0082231

**Published:** 2013-12-02

**Authors:** Coline C. Jaworski, Anaïs Bompard, Laure Genies, Edwige Amiens-Desneux, Nicolas Desneux

**Affiliations:** 1 Laboratoire Evolution et Diversité Biologique, UMR 5174, CNRS - Université Paul Sabatier, Toulouse, France; 2 Laboratoire Ecologie et Evolution, UMR 7625, Université Pierre et Marie Curie (Paris 6), Paris, France; 3 French National Institute for Agricultural Research (INRA), Sophia Antipolis, France; United States Department of Agriculture, Beltsville Agricultural Research Center, United States of America

## Abstract

Invasive pest species may strongly affect biotic interactions in agro-ecosystems. The ability of generalist predators to prey on new invasive pests may result in drastic changes in the population dynamics of local pest species owing to predator-mediated indirect interactions among prey. On a short time scale, the nature and strength of such indirect interactions depend largely on preferences between prey and on predator behavior patterns. Under laboratory conditions we evaluated the prey preference of the generalist predator *Macrolophus pygmaeus* Rambur (Heteroptera: Miridae) when it encounters simultaneously the local tomato pest *Bemisia tabaci* (Gennadius) (Hemiptera: Aleyrodidae) and the invasive alien pest *Tuta absoluta* (Meyrick) (Lepidoptera: Gelechiidae). We tested various ratios of local vs. alien prey numbers, measuring switching by the predator from one prey to the other, and assessing what conditions (e.g. prey species abundance and prey development stage) may favor such prey switching. The total predation activity of *M. pygmaeus* was affected by the presence of *T. absoluta* in the prey complex with an opposite effect when comparing adult and juvenile predators. The predator showed similar preference toward *T. absoluta* eggs and *B. tabaci* nymphs, but *T. absoluta* larvae were clearly less attacked. However, prey preference strongly depended on prey relative abundance with a disproportionately high predation on the most abundant prey and disproportionately low predation on the rarest prey. Together with the findings of a recent companion study (Bompard et al. 2013, *Population Ecology*), the insight obtained on *M. pygmaeus* prey switching may be useful for Integrated Pest Management in tomato crops, notably for optimal simultaneous management of *B. tabaci* and *T. absoluta*, which very frequently co-occur on tomato.

## Introduction

In ecosystems, species interact directly or indirectly resulting in both short-term effects on species abundance and density, and long-term effects on population dynamics [1-4]. Unlike direct interactions, indirect interactions are mediated by a third organism and may occur between organisms separated in time or space [1,5,6]. Generalist predators are likely to trigger indirect interactions among prey species owing to their capacity to attack different prey [7-9]. The nature or strength of predator-mediated indirect interactions may change over time, but are predicted to be generally positive at time scales shorter than the predator generation time (apparent mutualism or commensalism) [2,4,10]. The dispersion of predation pressure among multiple available prey species may result in increased prey population densities compared to densities in single prey systems. 

The nature of indirect interactions depends in part on predator preference [11,12]. Some of the prey characteristics that influence predator preference are nutritional quality of the prey and the ease of attack it presents [12]. Predation on prey of highest nutritive value increases the predator's fitness (higher survival, fecundity, etc...), although this prey may not be systematically preferred [12]. Capture success generally depends on prey mobility and access to a refuge (enemy-free space) [12,13]. 

Generalist hemipteran predators more frequently attack mobile prey: they are able to detect movements and hunt mobile prey [12,14], whereas they tend to move randomly on plants to find stationary prey [15]. When foraging, predators may also rely on some chemical cues to locate non-mobile prey such as semiochemicals resulting from prey oviposition or herbivore-induced plant volatiles (e.g. synomones) [16]. The tendency of a predator to choose a given prey type over another may change as the relative frequencies of the prey species in the predator’s environment change. Switching from one prey to the other occurs when the predator over-attacks the most abundant prey, and almost ignores the rarest one [17]. *Prey switching* has a stabilizing effect on prey populations as relatively scarce prey species are freed from predation and relatively common prey suffer it more frequently. Under this condition of disproportionate predation on more abundant prey, species neither go extinct nor proliferate [7,18]. This stabilizing effect of generalist predators on prey populations may have useful application for simultaneously managing multiple pest species in agro-ecosystems. Moreover, it may be a great help when developing biological control against invasive alien pest species. Invasive alien species generally have high capacities for proliferation; they may be strong competitors for resources and they may escape predation from their natural enemies when invading new regions [19,20]. Generalist predators, when switching between pests, may (i) help reduce overall pest pressure on crops and (ii) prevent new infestations by invasive alien pests [7,21].

We studied the predation behavior of the generalist mirid bug *Macrolophus pygmaeus* Rambur (Heteroptera: Miridae) feeding on two prey species, the local tomato pest *Bemisia tabaci* biotype Q (Gennadius) (Hemiptera: Aleyrodidae) and the invasive alien pest *Tuta absoluta* (Meyrick) (Lepidoptera: Gelechiidae). The South American tomato pinworm *T. absoluta* is a major pest on tomato [22]. It recently invaded Spain (2006) and quickly spread throughout the Afro-Eurasian continent [23]. The larvae cause dramatic yield decreases in tomato crops (up to 100%) by mining the leaves, stems and fruits of the plants [22]. *Bemisia tabaci* Biotype Q is a whitefly species from Europe [24-27] and a major pest in tomato crops causing direct and indirect (by vectoring viruses) damage [28,29]. *Macrolophus pygmaeus* is often used as a biocontrol agent against whiteflies (including *B. tabaci*). This predator also feeds on various other prey such as thrips, aphids, mites, and the eggs and larvae of Lepidoptera [30,31], notably *T. absoluta* [4,22,32]. It shows switching behavior when attacking whiteflies and other prey species [31]. *Macrolophus pygmaeus*, being native of Europe, has co-evolved with *B. tabaci*; it may show both preference and adaptation to this prey over recently invading alien species such as *T. absoluta*. Conversely, native prey may have evolved defense mechanisms against native predators that alien prey have not developed. As evolutionary naive prey, alien prey may suffer higher predation pressure than the native prey in the invaded area [20,33]. The predation behavior of *M. pygmaeus* when encountering both the local (*B. tabaci*) and invasive alien (*T. absoluta*) pests has not been described yet; it could affect efficacy of this predator as a biocontrol agent in tomato crops.

In this context, under laboratory conditions, we studied (i) the predation activity of *M. pygmaeus* in prey complex showing various ratios of local (*B. tabaci*) vs. alien (*T. absoluta*) prey numbers, (ii) the preference of *M. pygmaeus* for *B. tabaci* vs. *T. absoluta*, and (iii) potential *Prey switching* of *M. pygmaeus* between *B. tabaci* and *T. absoluta* when encountering both prey at various densities. 

## Materials and Methods

### Biological materials

The plants used in the experiments were tomato plants, *Solanum lycopersicum* L. cv. Marmande, grown in climatic chambers (23±1°C, 65±5% RH, 16L:8D) in individual plastic pots (diameter 26 cm). The prey *B. tabaci* and *T. absoluta* were reared on tobacco and tomato plants respectively, in separate cages, in a climatic chamber (23±1°C, 65±5% RH, 16L:8D). The predator *M. pygmaeus* was provided by Biotop© (InVivo AgroSolutions) and reared on tomato leaves (complemented with *Ephestia kuehniella* [Lepidoptera: Pyralidae] eggs) and maintained in growth chambers (23±1°C, 65±5% RH, 16L:8D). All predators used in the experiments lacked any previous experience of predation on *B. tabaci* or on *T. absoluta*, i.e. they were naive on these two prey. Each predator was isolated individually in a glass tube with a piece of tomato stem 24h before beginning each experiment.

### Experimental design

We studied the predatory behavior of *M. pygmaeus* in prey patches containing varying densities of *B. tabaci* and *T. absoluta*, on individual tomato plants (thereafter: microcosms), using a 2 x 2 x 4 factorial design. The first two-level treatment varied the predator stage tested (adult or juvenile). The second two-level treatment varied the presence of *T. absoluta* in the microcosms. The third four-level treatment varied the ratio between *B. tabaci* and *T. absoluta* in the prey complex introduced into the microcosms, while the total number of prey per microcosm remained constant at 40. The ratios tested of *B. tabaci* - *T. absoluta* were 40-0, 30-10, 20-20 and 10-30. No group was tested with *T. absoluta* as the sole prey because such a scenario would not be realistic for European tomato crops since whiteflies always infest the tomato crops before *T. absoluta* arrives. 

The prey and predator treatments chosen for the study were based on knowledge from the literature and from pilot experiments carried out in the lab. First, the predatory behavior of *M. pygmaeus* may change during its development; juveniles are assumed to have a lower satiety level than adults [30,34,35] and predatory behavioral pattern of Hemipteran juveniles can differ partially from those of adults [36]. Second, pilot experiments showed that predation on *B. tabaci* eggs by *M. pygmaeus* was quite marginal (< 5% of *B*. *tabaci* eggs attacked by the predator when providing 20, 30 or 40 eggs on a single leaflets, n=30 replicates per density tested). In addition, *M*. *pygmaeus* attacked very few *T*. *absoluta* old larvae (L3-L4) when compared to young larvae (L1-L2) or eggs of *T*. *absoluta* (< 3% of predation on L3-L4 during pilot experiments in Petri dishes, see also [37]). Therefore, the developmental stages of the prey used during the experiments were third nymph instars of *B*. *tabaci*, *T*. *absoluta* eggs, and *T*. *absoluta* young larvae (L1-L2). Third, at 25°C on tomato plants, the natural mortality of eggs and larvae of *T*. *absoluta* is low (2-15% depending on the *T*. *absoluta* stage considered, Table S1) and egg incubation and L1+L2 development times are very close (4.1±1.4 days and 4.8±0.5 days respectively) [22]. Therefore, when *T*. *absoluta* was used as prey, we used equal numbers of eggs and young larvae (L1-L2) in an attempt to create proportions of *T*. *absoluta* juvenile stages believed to occur naturally in tomato crops.

Following the design of previous studies [15,38], microcosms were created by placing a clear acetate cylinder over an individually potted tomato plant (4-week old plants with four fully expended leaves were used). Cylinders had a mesh (350 μm) top for ventilation. They were 35 cm high x 15 cm in diameter and sand was placed on the soil surface to provide a substrate into which the cylinder could be easily pushed to ensure a complete seal. All experiments were carried out at a temperature of 25±1°C, 70±5% RH and a 16L:8D photoperiod. For each *B. tabaci* - *T. absoluta* prey complex tested, crawlers of *B. tabaci* (first nymph instars, see [39]) were distributed equally among the leaves of the tomato plant with a fine brush, and nymph survival was checked 2 hours later under a microscope to ensure effective settlement of the nymphs. Plants were then placed in a climatic chamber for 7 days, sufficient time to allow *B. tabaci* nymphs to reach the third instar. After the 7-day period, *T. absoluta* eggs (laid for less than 10h [40]) and *T. absoluta* larvae (L1-L2) were deposited equally among the leaves of the tomato plant. The prey complex was allowed to settle for two hours on the plant before a single one predator (adult or juvenile) was introduced to each microcosm. The microcosms were then placed in growth chambers (25±1°C, 65±5% RH, 16L:8D). After 48h, the number of each prey type attacked by the predator was counted under a microscope.

Fifteen adult predators and 24 juvenile predators were exposed to each of the four *B. tabaci* - *T. absoluta* prey complexes. In all, 60 replicates were conducted with adult predators and 96 with juvenile predators. Data from microcosms in which the predator died or metamorphosed to an adult during the experiment were discarded from the analyses.

### Data analysis

Normality of datasets was assessed using a Shapiro-Wilk test, and statistical analyses were carried out with R software, version 2.14.1 (R Foundation for Statistical Computing). In order to characterize how the various treatments impacted *M. pygmaeus* predation, we used two types of analyses.

1 To assess the effect of (i) the predator stage, (ii) the presence of *T. absoluta* (in the prey complex), and (iii) the various *B. tabaci*-*T. absoluta* prey ratios (in the prey complex) on *M. pygmaeus* predation activity, the total number of prey attacked per microcosm was analyzed using a GLM analysis with the ‘‘predator stage’’, “*T. absoluta* presence”, and ‘‘*B. tabaci* - *T. absoluta* prey ratio” as main factors.
2 We used Manly’s modeling works [41,42] to assess (i) the preference of *M. pygmaeus* for either *B. tabaci* or *T. absoluta* in the microcosms, and (ii) *Prey* *switching* in *M. pygmaeus* when encountering various prey ratios (*B. tabaci* vs. *T. absoluta*) in the microcosms. In the general formula of Manly, a preference for a given prey is scored as a deviation in the number of individuals of a given prey type selected for a particular action from the number of this prey type available for the action. We used the number of prey attacked as the selected action and the number of prey per prey type in the microcosm as the number of available prey. As *M. pygmaeus* may feed differently on egg and L1-L2 of *T. absoluta* [37], we distinguished attacks occurring on *T. absoluta* larvae from those on *T. absoluta* eggs (as well as *B. tabaci* nymphs). Manly’s *βj* of the *j*th prey type for predation event (with three prey types being considered) was estimated using the equation (*18*) of Manly et al. [42]:

$\beta j=\frac{\ln\;(rj/Aj)}{\sum_{i=1}^{n}\ln\;(ri/Ai)}$ *j* =1, 2, 3

*Ai* was the number of individuals of a given prey type *i* available for predation by *M. pygmaeus* ($\sum_{i=1}^{3}Ai$= total number of prey available for predation) and *ri* was the number of a prey type *i* that have not been attacked (with *xi* the number of a prey type *i* attacked and xi+*ri*=*Ai*). The number of prey types was *n=3* and *βj* = 1/ *n* when prey were chosen randomly (for all *j*). The decrease of available prey as predation occurred during the experiment was approximated with the use of logarithms [41,42]. The preference of *M. pygmaeus* for a given prey type over other ones (per prey complex tested, i.e. per *B. tabaci - T. absoluta* ratio) was tested by comparing Manly’s Beta values among *T. absoluta* eggs, *T. absoluta* larvae and *B. tabaci*; we used an ANOVA followed by a Tukey’s post hoc test for multiple comparisons. In addition, the occurrence of a *Prey switching* in *M. pygmaeus* was tested using a Student’s t-test that compared estimated *βj* values from expected values [31,41,42]. 

## Results

### Predation activity

**Figure 1:**
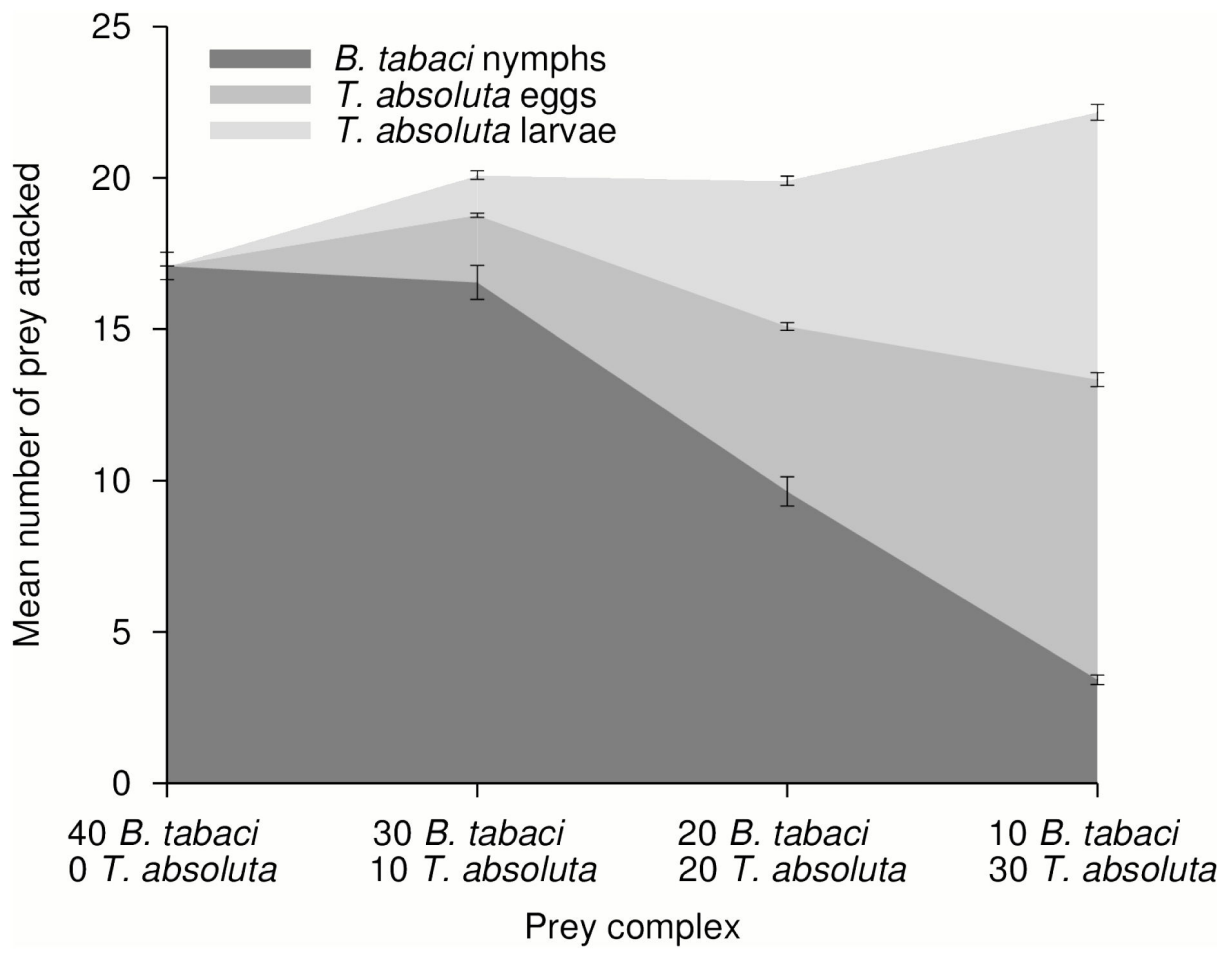
Predation of *B. tabaci* (nymphs) and *T. absoluta* (eggs and larvae) by *M. pygmaeus* adult predators in various Prey complex. Mean number (±SEM) of prey attacked by *M. pygmaeus* adult predators per prey type and as function of the various *B. tabaci* and *T. absoluta* prey ratio (Prey complex) tested in the microcosms. Dark grey: predation on *B. tabaci* nymphs; medium grey: predation on *T. absoluta* eggs; light grey: predation on *T. absoluta* larvae.

**Figure 2:**
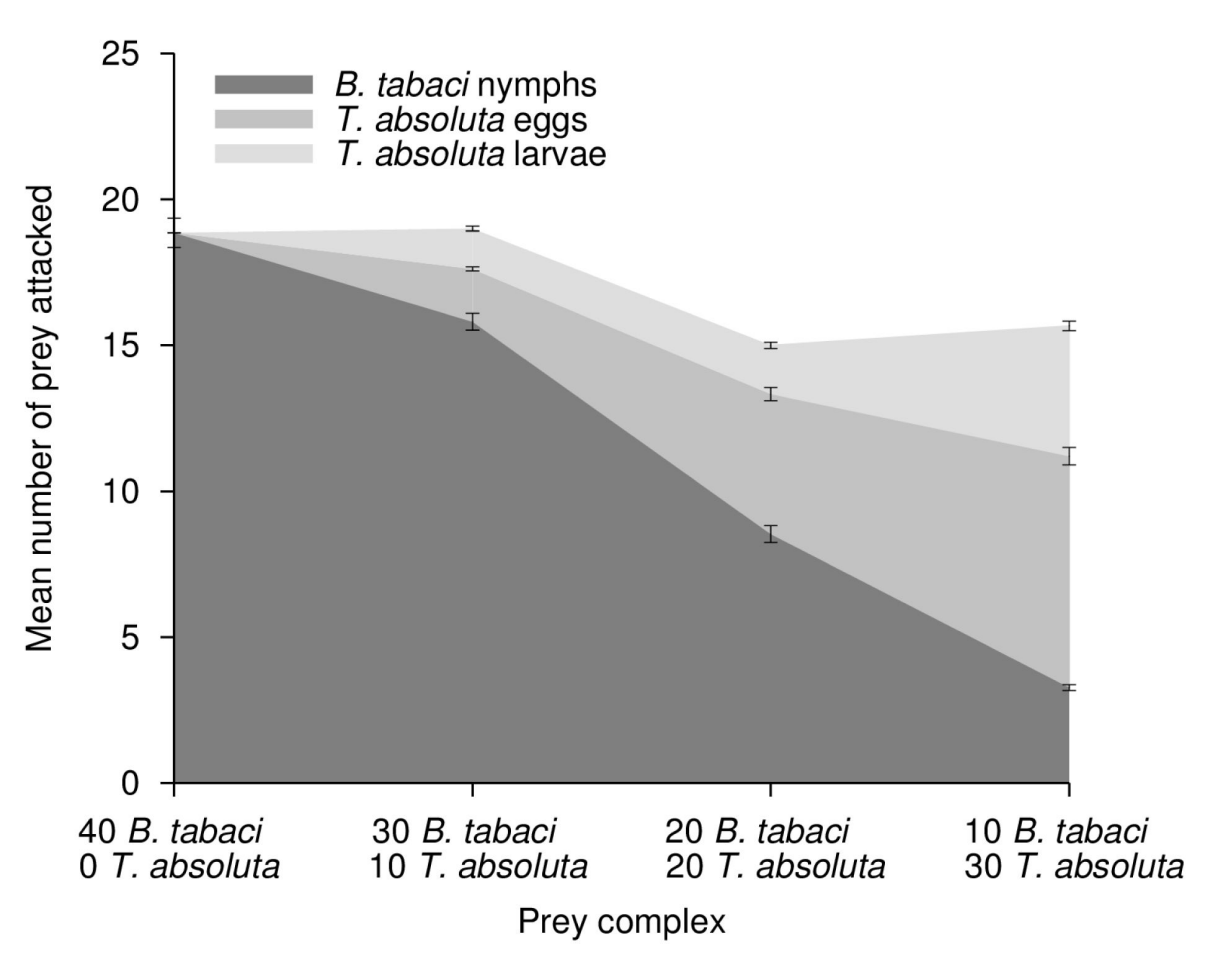
Predation of *B. tabaci* (nymphs) and *T. absoluta* (eggs and larvae) by *M. pygmaeus* juvenile predators in various Prey complex. Mean number (±SEM) of prey attacked by *M. pygmaeus* juvenile predators per prey type and as function of the various *B. tabaci* and *T. absoluta* prey ratio (Prey complex) tested in the microcosms. Dark grey: predation on *B. tabaci* nymphs; medium grey: predation on *T. absoluta* eggs; light grey: predation on *T. absoluta* larvae.

The statistical results of the GLM analysis are summarized in Table 1. The total predation activity of *M. pygmaeus* in the microcosms (i.e. all prey attacked, pooled per microcosm) varied significantly between the predator stages (significant ‘Predator stage’ factor); there was higher predation by adults than by juveniles (Figures 1 and 2). By contrast, neither the presence of *T. absoluta* nor the prey ratio (*B. tabaci* – *T. absoluta*) in the microcosm affected the predation activity of *M. pygmaeus* (non significant ‘*Tuta absoluta*’ and ‘Prey ratio’ factors). However, the ‘Predator stage’ and ‘*Tuta absoluta*’ factors did interact significantly; suggesting that the effect of predator stage on predation activity was function of the presence or not of *T. absoluta*. The presence of *T. absoluta* in the prey complex led to an increased predation for adults (Figure 1) whereas it led to a reduced predation activity for juveniles (Figure 2). In addition, impact of predator stage was also function of the *B. tabaci* – *T. absoluta* ratio (significant interaction between ‘Predator stage’ and ‘Prey ratio’ factors). When the prey ratio was biased toward *T. absoluta*, the predation activity of adult predators increased by up to 30% (Figure 1). By contrast, an increased proportion of *T. absoluta* in the prey ratio led to a reduction of predation activity by juveniles (Figure 2); it decreased by up to 20.5% when *B. tabaci* represented only 0.25 of prey available in the microcosms. 

**Table 1:** Statistics from the generalized linear model used to analyze the number of prey attacked by *M. pygmaeus* in microcosms as function of predator stage (adults vs. juveniles, ‘Predator stage’ factor), as function of the presence or not of *T. absoluta* in the microcosms (‘*Tuta absoluta*’ factor), and as function of the various *B. tabaci* - *T. absoluta* prey ratio tested (‘Prey ratio’ factor).

Source of variation	Degrees of freedom	Chi-square	p-value
Predator stage	1	10.38	0.001
*Tuta absoluta*	1	1.04	0.308
Prey ratio	3	5.63	0.131
Predator stage x *Tuta absoluta*	1	15.14	< 0.001
Predator stage x Prey ratio	3	20.67	< 0.001

### Predator preference

**Figure 3:**
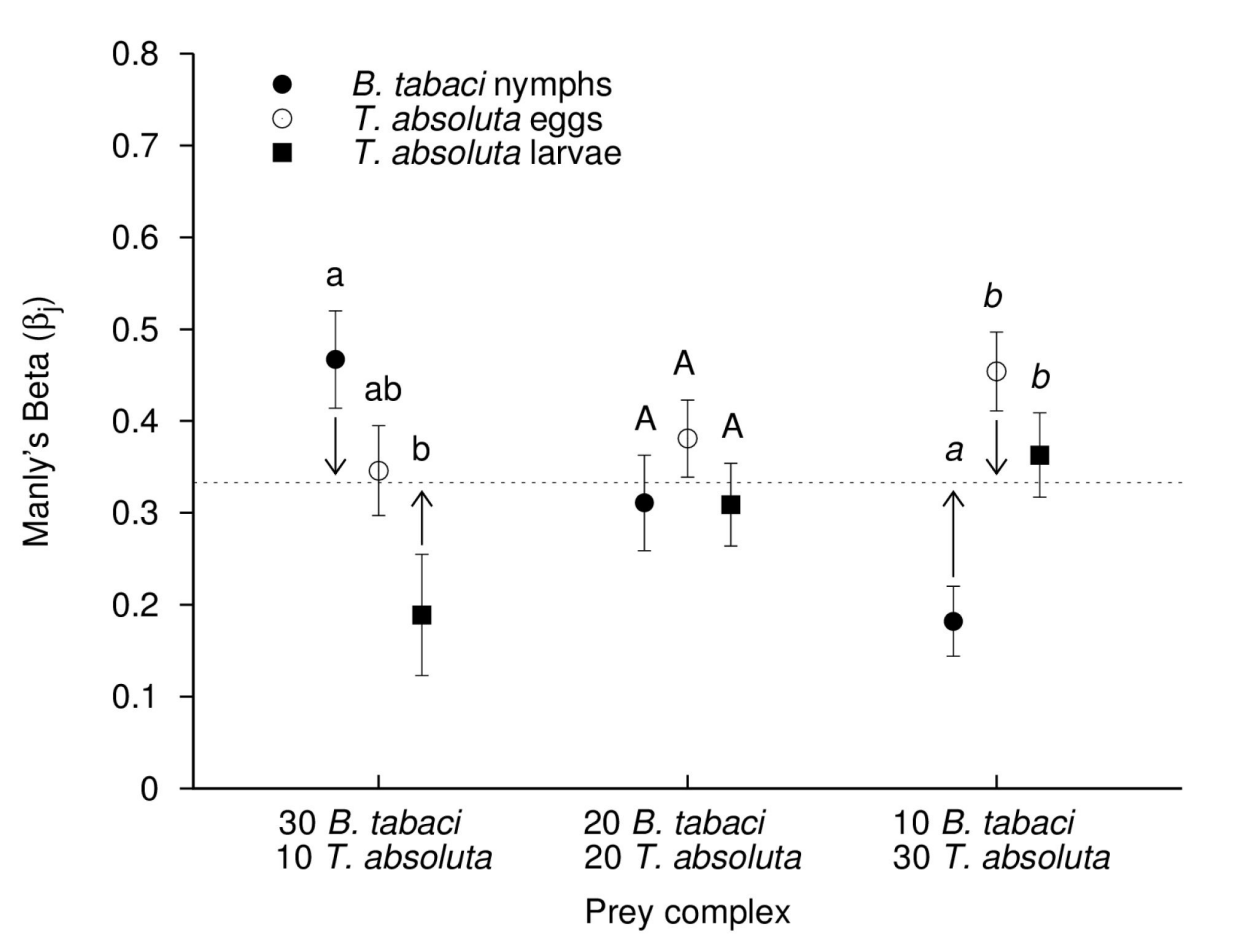
Prey preference of *M. pygmaeus* adult predators (based on Manly’s Beta values) depending on initial ratio among prey. Manly’s Beta values (± SE) for *M. pygmaeus* adult predators in three-prey patches (*B. tabaci* nymphs, *T. absoluta* eggs and *T. absoluta* larvae) with various *B. tabaci* – *T. absoluta* prey ratios (Prey complex). Dotted line represents the expected *βj* value against which calculated *βj* values for each prey are compared (Student’s t-test, significance difference with expected *βj* values are indicated by arrows, at the 0.05 level). Different letters for a given *B*. *tabaci* – *T*. *absoluta* prey ratio indicate significantly different *βj* values between the three prey types (*P* > 0.05, ANOVA with Tukey’s post-hoc analysis).

**Figure 4:**
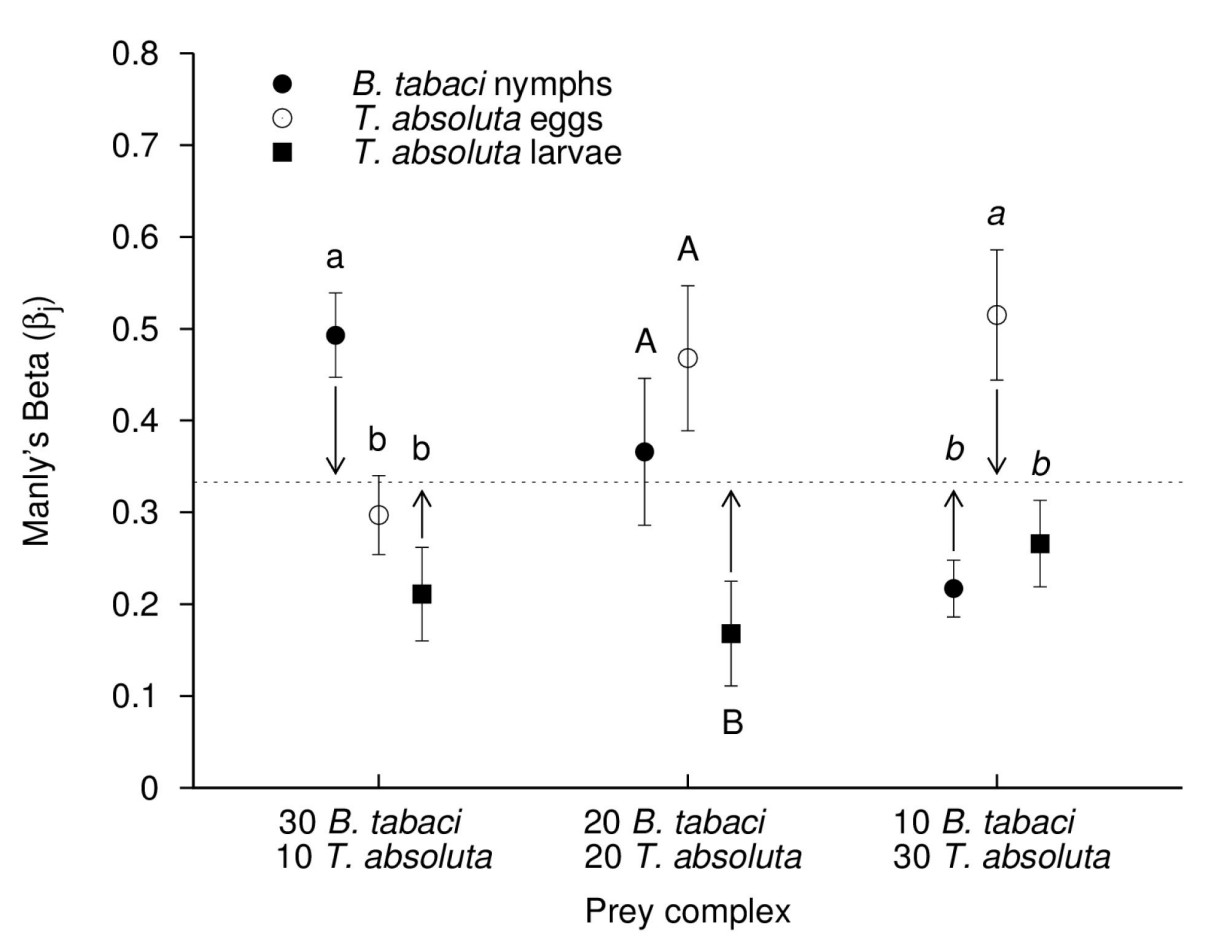
Prey preference of *M. pygmaeus* juvenile predators (based on Manly’s Beta values) depending on initial ratio among prey. Manly’s Beta values (± SE) for *M. pygmaeus* juvenile predators in three-prey patches (*B. tabaci* nymphs, *T. absoluta* eggs and *T. absoluta* larvae) with various *B. tabaci* – *T. absoluta* prey ratios (Prey complex). Dotted line represents the expected *βj* value against which calculated *βj* values for each prey are compared (Student’s t-test, significance difference with expected *βj* values are indicated by arrows, at the 0.05 level). Different letters for a given *B*. *tabaci* – *T*. *absoluta* prey ratio indicate significantly different *βj* values between the three prey types (*P* > 0.05, ANOVA with Tukey’s post-hoc analysis).

The assessment of predator preference was based on the analyses of Manly’s Beta values (*βj*). For all *B. tabaci*-*T. absoluta* prey ratios tested, *B. tabaci* was the significantly preferred prey in half of the cases. It was the preferred prey for adult predators when tested at the 30-10 *B. tabaci* – *T. absoluta* ratio (Figure 3, F_2,32_ = 6.024, *P* = 0.008) and the preferred one for juvenile predators when tested at the 30-10 and 20-20 *B. tabaci* – *T. absoluta* ratio (Figure 4, F_2,47_ = 9.622, *P* < 0.001 and F_2,44_ = 4.409, *P* = 0.018, respectively). Similar situations occurred for *T. absoluta* eggs, except that this prey type was preferred in two cases by juvenile predators (at 20-20 and 10-30 *B. tabaci* – *T. absoluta* ratio, Figure 4, F_2,44_ = 4.409, *P* = 0.018 and F_2,44_ = 8.726, *P* = 0.001, respectively), and only once for adult predators (at 10-30 *B. tabaci* – *T. absoluta* ratio) (Figure 3, F_2,35_ = 10.667, *P* < 0.001). When compared to other prey types, *T. absoluta* larvae were the preferred prey only when adult predators were in microcosms containing the 10-30 *B. tabaci* – *T. absoluta* ratio. By contrast, for juvenile predators *T. absoluta* larvae were less preferred for all the tested prey ratios.

### Prey switching in *Macrolophus pygmaeus*

When exposed to the various *B. tabaci* - *T. absoluta* prey ratios in the microcosms, *Prey switching* was observed in both adult and juvenile predators; they over-attacked the most abundant prey when the prey complex was either biased toward *B. tabaci* or toward *T. absoluta* (Figures 3 and 4). More specifically, when *B. tabaci* was the predominant prey (30-10 *B. tabaci*-*T. absoluta* ratio) the calculated *βj* values for *B. tabaci* were significantly higher than the expected *βj* values (predator adults: Figure 3, *t* = 2.514, df = 11, *P* = 0.036; predator juveniles: Figure 4, *t* = 3.561, df = 15, *P* = 0.003). By contrast at that prey ratio, the *βj* values for *T. absoluta* larvae were significantly lower than the expected *βj* values for this prey type (predator adults: Figure 3, *t* = - 2.139, df = 11, *P* = 0.045; predator juveniles: Figure 4, *t* = -2.363, df = 15, *P*=0.032). In a similar way, when *T. absoluta* was the predominant prey, i.e. at ratio 10-30 *B. tabaci*-*T. absoluta*, the calculated *βj* values for *B. tabaci* were significantly lower than the expected *βj* values (predator adults: Figure 3, *t* = - 3.902, df = 11, *P* = 0.002; predator juveniles: Figure 4, *t* = - 3.603, df = 14, *P* = 0.003). However, the *βj* values for *T. absoluta* eggs were significantly higher than the expected (*βj* values for this prey type) at the 10-30 *B. tabaci* - *T. absoluta* prey ratio (predator adults: Figure 3, *t* = 2.873, df = 11, *P* = 0.015; predator juveniles: Figure 4, *t* = 2.584, df = 14, *P*=0.022). When *B. tabaci* and *T. absoluta* were evenly present in the microcosms (ratio 20-20 *B. tabaci*-*T. absoluta*), no prey was over- or under-attacked by the predator (all *P* ≥ 0.102) except for *T. absoluta* larvae that were les attacked by predator juveniles than predicted by the expected *βj* value (Figure 4, *t* = - 2.853, df = 14, *P*=0.013). 

## Discussion

Our study confirmed the predation of *M. pygmaeus* on the local pest *B. tabaci* and the invasive pest *T. absoluta* as previously reported by Bompard et al. [4]. We further demonstrated that, in the short term, preference toward a given prey type depended on the ratio between the prey species *B. tabaci* and *T. absoluta* on the tomato plant. In addition, we showed that the presence of *T. absoluta* on the plant affected the predation activity of *M. pygmaeus* in opposite ways for predator adults and juveniles: the presence of *T. absoluta* induced an increase of predation by predator adults whereas it led to decreased predation by juveniles. That decrease for juveniles was mainly due to low predation on *T. absoluta* larvae; the more *T. absoluta* larvae present in the prey complex, the lower the overall predation activity by predator juveniles. We demonstrated that *M. pygmaeus* can exhibit *Prey switching* [17] when foraging in areas where both *T. absoluta and B. tabaci* are present in varying proportion; the predator consistently showed disproportionately high and low predation on the most abundant and the rarest prey, respectively.

Overall, the predation activity of *M. pygmaeus* juveniles was lower than predation by adults, as already highlighted in a previous study [30]. We believe this may result from the limited ability of juveniles to attack *T. absoluta* larvae. We noted that adult and juvenile predators attacked a similar number of *B. tabaci* nymph when the nymph was the sole prey in the microcosms (comparison of adult and juvenile predators for the prey ratio 40-0 *B. tabaci* – *T. absoluta* in Figures 1 and 2). This lower predation activity of juveniles on *T. absoluta* larvae may be due to the prey size relative to the predator size, which can impact prey preference in generalist predators [43]. This possibility is consistent with the increased predation activity recorded for predator adults when *T. absoluta* larvae were present in the microcosms since predator adults are bigger than juveniles and more able to attack bigger prey. Morphological characteristics of *M. pygmaeus* juveniles, such as a shorter rostrum than adults, may also explain the low predation on *T. absoluta* larvae since juveniles may not be able to attack *T. absoluta* that are hidden inside mines in tomato leaves; attacking these larvae requires piercing both the tomato leaf and larvae cuticle. In our study, *M. pygmaeus* juveniles took likely more time to attack *T. absoluta* larvae than to attack *B. tabaci* nymphs and *T. absoluta* eggs. The presence of *T. absoluta* larvae in a prey patches may lead to an overall reduced efficiency of *M. pygmaeus* juveniles as predators. By contrast, *M. pygmaeus* adults showed increased predation activity when *T. absoluta* larvae were present in the prey patch.

 When considering the *M. pygmaeus* population as a whole (i.e. adults + juveniles) the net outcome of the reduced predation activity of juveniles coupled with the increased predation activity of adults is unclear. However, a previous study demonstrated the positive effect of *T. absoluta* presence on the biocontrol of *B. tabaci* by *M. pygmaeus* in tomato greenhouses [4]. This suggests that the positive effect on adult predation activity might overwhelm the negative effect on juvenile activity. In our study *M. pygmaeus* juveniles did show an active predation behavioral pattern despite *T. absoluta* larvae presence; they may still participate noticeably in pest regulation on the tomato plants despite presence of *T. absoluta* larvae.

Prey preference in generalist predators is driven by trade-offs among various mechanisms, notably the ease of attacking different prey as well as the differing nutritional value of the various prey to the predator [12]. The ease of attacking a given prey depends on various characteristics, the main factors are (i) the capacity to detect prey, (ii) how easy the predator can access to prey, (iii) the defenses exhibited by prey against predators, and (iv) the capacity to effectively feed on prey [15,44-46]. Hemipteran predators are able to forage specifically for mobile prey by detecting prey movements, whereas they forage for non-mobile prey through random movements both on and among plants that may host prey [14,15,47]. In our study, the only mobile prey was *T. absoluta* larvae. However, *T. absoluta* larvae spend most of their time feeding and moving in leaf mines where they are less accessible to predators [22]. This possibility to benefit from spatial refuges within the plant could explain the lower predation on this prey type [48]. A higher predation rate on eggs than on larvae of *T. absoluta* has already been reported in a previous study [37]; however this study was not based on choice tests while our study further documented *M. pygmaeus* preference between *T. absoluta* eggs and larvae in a choice scenario.

Several factors may explain a possible preference of the predator for *T. absoluta* eggs over *B. tabaci* nymphs. This preference may occur because handing time (i.e. time between first encounter with a prey and the end of predation event, see [49]) of *T. absoluta* egg by *M. pygmaeus* is much faster than on *B. tabaci* nymph (20-30 min. and 4-5 min., respectively, Jaworski CC, personal observation). In addition, *T. absoluta* is a lower quality food than *B. tabaci* for *M. pygmaeus*; during a pilot experiment, we observed lower fecundity and longevity of *M. pygmaeus* fed on *T. absoluta* eggs than when fed on *B. tabaci* nymphs (Figure S1), and a recent study also reported poor nutritional value of *T. absoluta* eggs for *M. pygmaeus* [50]. Moreover, we suppose the size of the two prey to be of low importance because they are in the same size range (400µm. for *T. absoluta* eggs vs. 500µm for *B. tabaci* nymphs [51,52]). 

In our study, the absence of a clear preference of *M. pygmaeus* between *T. absoluta* eggs and *B. tabaci* nymphs highlighted the importance of *Prey switching* [17] in the predation behavior of this predator. Predation preference depended strongly on the relative abundances of the prey species, with a disproportionately high predation on the most abundant prey and a disproportionately low predation on the rarest prey. Such *Prey switching* had been previously reported for *M. pygmaeus* preying upon *B. tabaci* and the spider mites [31] and it is thought to be exhibited by many generalist predators [17]. Clumped and patched prey distributions are common in natural conditions, leading to spatial heterogeneities and context-dependent predation behaviors. *Prey switching* can enable predators to maximize food intake by increasing foraging time in patches showing high density of one prey type [53]; *M. pygmaeus* likely benefits from such adaptative behavior when foraging in crops where *B. tabaci* and *T. absoluta* co-occur. 

Our study confirmed the ability of *M. pygmaeus* to attack *T. absoluta* (already suggested by previous results under greenhouse and laboratory conditions, respectively [4,37,46]) and demonstrated that the predator is able to switch between the alien and the local prey when foraging in habitats hosting both prey. However, the low nutritive quality of *T. absoluta* for *M. pygmaeus* ([50] and Figure S1) tempers any conclusion about its potential to be a good candidate for the biological control of *T. absoluta* in tomato crops (at least not as the key natural enemy of *T. absoluta* in tomato crops if not included in a broader IPM program; see [32]). Using *M. pygmaeus* as a biocontrol agent against *T. absoluta* would require the presence of an alternate prey to sustain growth of the predator population. In a situation requiring simultaneous control of both *B. tabaci* and *T. absoluta*, the presence of *T. absoluta* might disrupt the biocontrol of *B. tabaci* in the short term because *M. pygmaeus* would spend time attacking *T. absoluta* eggs and larvae (larvae to a lesser extent). Greenhouse experiments showed a transient disruption of the predation on *B. tabaci* by *M. pygmaeus* when *T. absoluta* was present in the tomato crop, but the control of *B. tabaci* populations was enhanced in the long terms [4]. The *Prey switching* exhibited by *M. pygmaeus* when encountering both *B. tabaci* and *T. absoluta* prey might prevent fast population growth of either of the two prey (as stressed in other studies on generalist predators [7,17,54]). If *Prey switching* is maintained at larger scales (agro-ecosystem) it may help regulating both prey populations simultaneously to low densities. *Macrolophus pygmaeus* could be useful for IPM programs since the probability for both *B. tabaci* and *T. absoluta* to be present simultaneously in tomato crops is high in numerous areas cropped with tomato in Afro-Eurasia [22,23]. The presence of *B. tabaci* on tomato crops early in the season may help *M. pygmaeus* populations to establish prior to *T. absoluta* infestation. The knowledge gained during our studies ([4] and the present study) and previous theoretical works on *Prey switching* suggest that *M. pygmaeus* may not attack *T. absoluta* before this prey becomes abundant in the field [17,54]. However, a small primary infestation of tomato plants by *T. absoluta* may rapidly lead to very high population densities owing to its high reproduction rate [22] and the capacity of *M. pygmaeus* to effectively limit *T. absoluta* population growth could be exceeded [4,55]. In addition, the fact that *T. absoluta* is a low quality food for *M. pygmaeus* may be detrimental in the long term to value of the biocontrol service provided by *M. pygmaeus*. High rates of attacks on prey without a significant increase in predator fitness have already been reported for Hemipteran predators in laboratory and field studies [8,15] and such predation behavior may lead to a relatively good control of *T. absoluta* by *M. pygmaeus* in the short term. However, the predator's biocontrol efficacy may be reduced in the long term by its lower population growth when consuming prey of poor nutritive value. *Prey switching* in *M. pygmaeus* when attacking *B. tabaci* and *T. absoluta* needs to be further assessed at larger scales including direct field observations along with an assessment of the impact of poor quality food on the ability of this predator to provide useful biocontrol services [4,56].

## Supporting Information

Table S1 **Natural mortality of *T. absoluta* under laboratory conditions at the various instars.** Survival of *T. absoluta* from egg to adulthood was evaluated by placing *T. absoluta* eggs individually (n=60) in aerated plastic boxes (diameter: 110 cm, height: 2 cm, with a circular opening made of nylon mesh netting, 350 mm^2^) together with a single tomato leaf. The tomato steam was inserted in a tube containing water. Boxes were placed in rearing chambers (23±1°C, 65±5% RH, 16L:8D) and we followed *T. absoluta* development until death or adulthood.
(PDF)

Figure S1 **(**A**) Mean longevity (± SEM) of *Macrolophus pygmaeus* adult (in days) and (**B**) mean daily fertility (± SEM) of *M. pygmaeus* (offspring per day per female).** Longevity and fecundity were evaluated by placing *M. pygmaeus* adults individually (n=40) in aerated plastic boxes (diameter: 110 cm, height: 2 cm, with a circular opening made of nylon mesh netting, 350 mm) together with a single tomato leaf (replaced every day for further assessment of offspring production). The tomato steam was inserted in a tube filled with water. Insects were provided daily with the prey *ad* *libitum* (*B. tabaci* nymphs and *T. absoluta* eggs) accordingly to respective treatment. Boxes were placed in rearing chambers (23±1°C, 65±5% RH, 16L:8D). Histograms bearing different letters are significantly different to each other (*P* < 0.05, GLM followed by a Tukey’s post-hoc test). GLM results: (A) Chi-square = 6.60, df = 2, *P* = 0.037; (B) Chi-square = 13.26, df = 2, *P* = 0.001.
(PDF)
