# Supplementary material for: Preference and Prey Switching in a Generalist Predator Attacking Local and Invasive Alien Pests
Source: PLoS One. 2013 Dec 2;8(12):e82231. doi: 10.1371/journal.pone.0082231 (PMC3846826; doi:10.1371/journal.pone.0082231)
Supplement: Table S1 — Natural mortality of T. absoluta under laboratory conditions at the various instars. Survival of T. absoluta from egg to adulthood was evaluated by placing T. absoluta eggs individually (n=60) in aerated plastic boxes (diameter: 110 cm, height: 2 cm, with a circular opening made of nylon mesh netting, 350 mm2) together with a single tomato leaf. The tomato steam was inserted in a tube containing water. Boxes were placed in rearing chambers (23±1°C, 65±5% RH, 16L:8D) and we followed T. absoluta development until death or adulthood. (PDF) [file pone.0082231.s001.pdf]

Table S1 (Jaworski CC, Bompard A, Genies L, Amiens-Desneux E, Desneux N, 2013, Preference and prey switching in a generalist predator attacking local and invasive alien pests. PLoS ONE. doi:10.1371/journal.pone.0082231.)

Tuta absoluta stage	Egg	L1	L2	L3	L4	Pupae
Mortality (%)	1.7	15.2	4.0	6.2	6.6	2.5
Cumulated mortality (%)	1.7	17.0	21.0	27.2	33.8	36.3
