# Supplementary material for: Preference and Prey Switching in a Generalist Predator Attacking Local and Invasive Alien Pests
Source: PLoS One. 2013 Dec 2;8(12):e82231. doi: 10.1371/journal.pone.0082231 (PMC3846826; doi:10.1371/journal.pone.0082231)

Figure S1 (Jaworski CC, Bompard A, Genies L, Amiens-Desneux E, Desneux N, 2013, Preference and prey switching in a generalist predator attacking local and invasive alien pests. PLoS ONE. doi:10.1371/journal.pone.0082231.)

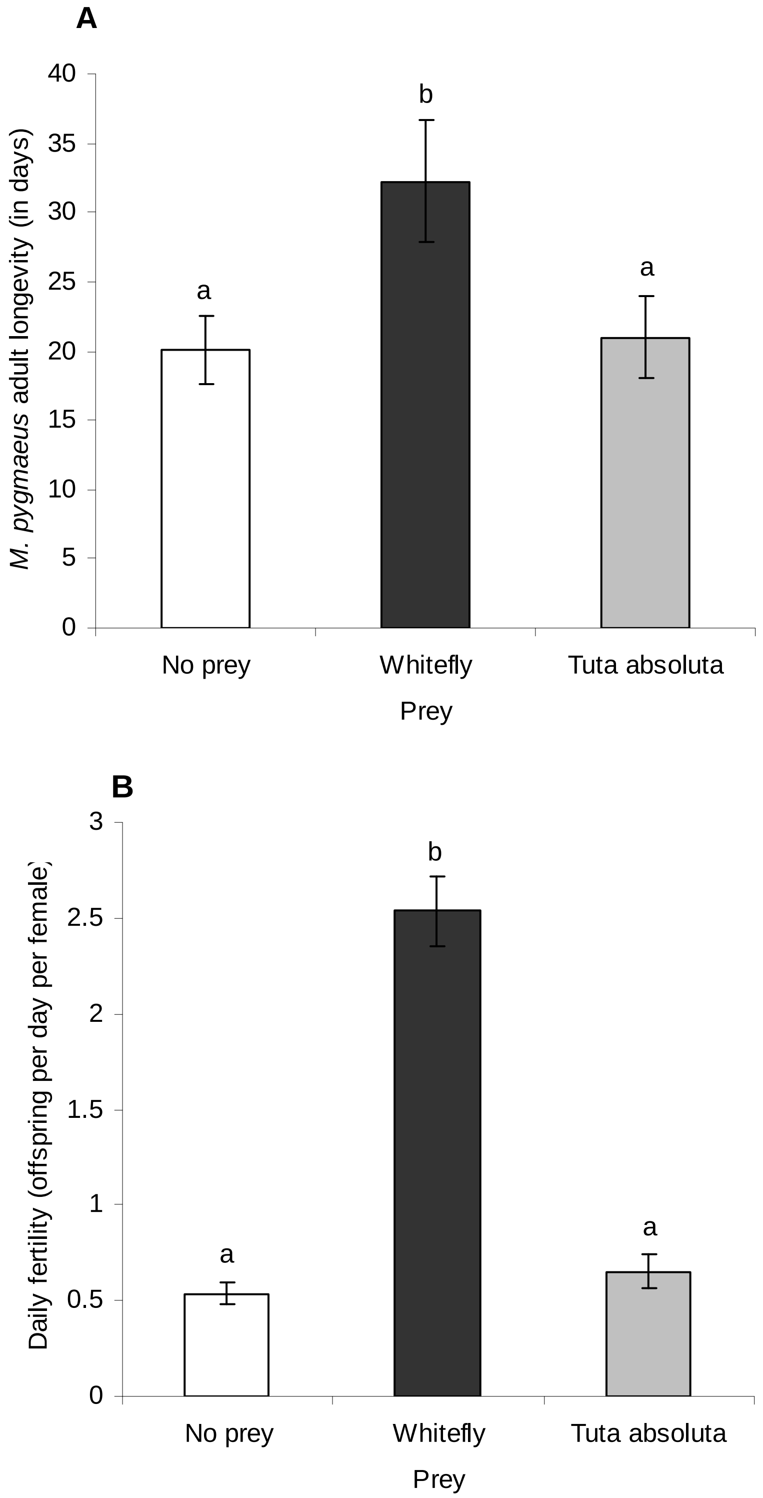

Supplement: Figure S1 — (A) Mean longevity (± SEM) of Macrolophus pygmaeus adult (in days) and (B) mean daily fertility (± SEM) of M. pygmaeus (offspring per day per female). Longevity and fecundity were evaluated by placing M. pygmaeus adults individually (n=40) in aerated plastic boxes (diameter: 110 cm, height: 2 cm, with a circular opening made of nylon mesh netting, 350 mm) together with a single tomato leaf (replaced every day for further assessment of offspring production). The tomato steam was inserted in a tube filled with water. Insects were provided daily with the prey ad libitum (B. tabaci nymphs and T. absoluta eggs) accordingly to respective treatment. Boxes were placed in rearing chambers (23±1°C, 65±5% RH, 16L:8D). Histograms bearing different letters are significantly different to each other (P < 0.05, GLM followed by a Tukey’s post-hoc test). GLM results: (A) Chi-square = 6.60, df = 2, P = 0.037; (B) Chi-square = 13.26, df = 2, P = 0.001. (PDF) [file pone.0082231.s002.pdf]
